# Designed Structures of Interdigital Electrodes for Thin Film SAW Devices

**DOI:** 10.3390/mi14101929

**Published:** 2023-10-14

**Authors:** Yicong Qian, Yao Shuai, Chuangui Wu, Wenbo Luo, Xinqiang Pan, Wanli Zhang

**Affiliations:** 1 Chongqing Institute of Microelectronics Industry Technology, University of Electronic Science and Technology of China, Chongqing 401332, China; 13700478616@163.com (Y.Q.); cgwu@uestc.edu.cn (C.W.); luowb@uestc.edu.cn (W.L.); panxinqiang@uestc.edu.cn (X.P.); wlzhang@uestc.edu.cn (W.Z.); 2 School of Electronic Science and Engineering, University of Electronic Science and Technology of China, Chengdu 611731, China

**Keywords:** SAW resonator, interdigital electrodes, transverse modes, impedance ratio, PDT structure, GLONASS filter

## Abstract

This paper studied the impact of the microstructure of interdigital electrodes on the performance of surface acoustic wave (SAW) resonators and proposed an innovative piston, dummy finger and tilt (PDT) structure, which was then applied to the GLONASS L3 band filters. Through the adoption of 3D finite element simulation (FEM), photolithography, and testing on an incredible high-performance surface acoustic wave (I.H.P. SAW) substrate, it is concluded that the total aperture length is 20*T* (*T* is period), resulting in a more optimal resonator performance; changing the width and length of the piston can suppress transverse modes spurious, but it does not enhance impedance ratio; to further improve the quality of the SAW resonator, the proposed PDT structure has been experimentally proven to not only effectively suppress transverse modes spurious but also possess a high impedance ratio. By utilizing a PDT structure within a “T + π” topology circuit, we successfully designed and manufactured a GLONASS L3 band filter with a bandwidth of 8 MHz and an insertion loss of 3.73 dB. The design of these resonators and filters can be applied to the construction of SAW filters in similar frequency bands such as BeiDou B2 band or GPS L2/L5 band.

## 1. Introduction

**Figure 1:**
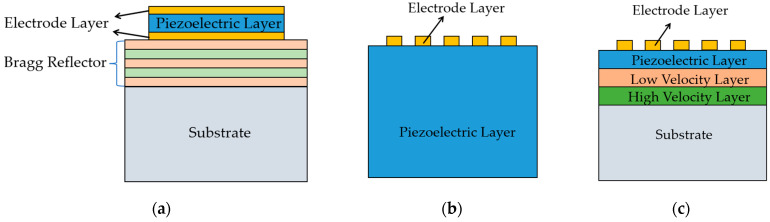
Schematic diagram of BAW/SAW resonators. (**a**) SMR type BAW resonator; (**b**) SAW bulk resonator; (**c**) I.H.P. SAW resonator.

Surface acoustic wave and bulk acoustic wave (BAW) resonators are broadly utilized in the filter units of the RF front-end of wireless communication due to their small package area and high-quality factor (Q) value [1,2,3,4]. BAW resonators have the ability to modify the working frequency by adjusting the thickness of the piezoelectric film, making them extensively used in frequency bands above 3.5 GHz [5,6]; the schematic diagram is shown in Figure 1a. BAW resonators have several advantages such as concentrated acoustic energy, low loss, and high-power capacity [7], but they require a long development period, involve numerous production process steps, and are ultimately costly. In comparison to BAW resonators, traditional SAW bulk resonators have a straightforward manufacturing process and are priced affordably. A traditional SAW bulk resonator consists of a whole piece of piezoelectric material and interdigital electrodes above it [8,9]. As shown in Figure 1b, it has the characteristics of low production difficulty and cost and has been widely used in the design of 4G RF circuits. Nevertheless, due to the limitations of photolithography technology, the width of the interdigital electrodes cannot be further reduced, resulting in its application only in frequency bands less than 2 GHz. In addition, traditional SAW bulk resonators also have the drawbacks of a large temperature coefficient of frequency (TCF) [10]. To solve the problem of SAW bulk resonators’ drawbacks, an I.H.P. SAW resonator structure has emerged [11,12], as shown in Figure 1c. This resonator boasts the advantages of limiting the surface energy effect, having a high-quality factor value, excellent frequency stability, superior heat dissipation performance, and a wider range of applications in resonator frequency compared to traditional SAW bulk resonators. However, the I.H.P. SAW resonator possesses a defect that SAW bulk resonators do not have, which is the presence of transverse modes spurious [13].

In recent years, researchers have discovered that there are two primary methods for suppressing transverse modes spurious. One method involves tilting the resonator at an angle in the direction of SAW propagation. Although the transverse modes spurious are suppressed after the resonator is tilted, its Q value decreases compared to the non-tilted resonator [14]. The other method involves adding a piston structure to the dummy finger [15,16] which can suppress the transverse modes spurious while maintaining the Q value without decreasing. In a piston structure, the aperture gap, the piston width and length, and the dummy finger of the resonator exert varying degrees of influence on transverse modes’ suppression ability and Q value. Hence, how to further improve the performance of I.H.P. SAW resonators by adding the piston structure to the dummy fingers has become an urgent issue in current research.

In order to design GLONASS L3 band filters with low insertion loss and an extremely narrow bandwidth, it is necessary to require SAW resonators to have a very high impedance ratio and strong ability to suppress transverse modes spurious. Based on the advantages of piston and tilt structures, an innovative PDT structure is proposed, which not only enables resonators to achieve a high impedance ratio of over 90 dB at periods of 2.92 μm and 3.28 μm, but also effectively suppresses transverse modes spurious. These characteristics make it suitable for designing GLONASS L3 band filters with an extremely narrow bandwidth and low insertion loss.

This article investigates the effect of the total aperture length, piston width, piston length and a PDT structure on the performance of the interdigital electrodes on LT/SiO_2_/Poly Si/Si substrate through FEM and experiments. In addition, a PDT structure is applied to the design and production of a very narrow bandwidth and low insertion loss GLONASS L3 band filters.

## 2. Piston Structure of I.H.P. SAW Resonator

The interdigital electrodes are the fundamental component of the SAW resonator. In the SAW resonator, the interdigital electrode is mainly used to complete the mutual conversion between electrical and acoustic energy and limit the propagation direction of surface acoustic waves. The performance of the SAW resonator directly determines the performance of the SAW filter. By appropriately designing the shape of each interdigital electrode, such as its structural size, relative position, and degree of overlap, or by making appropriate changes in the direction of SAW propagation, the expected resonant frequency can be obtained accordingly, thereby achieving the goal of electrical signal processing.

**Figure 2:**
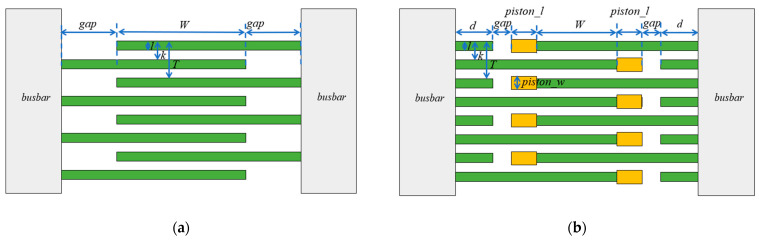
Interdigital electrode structure. (**a**) Uniform interdigital electrode; (**b**) piston mode interdigital electrode.

The structures of interdigital electrodes are diverse; Figure 2a shows the simplest and most commonly used interdigital electrode structure. Among them, *l*, *k*, *T*, *W*, and *gap* represent the width of the interdigital electrode finger, the length of the interdigital electrode finger width plus the distance between fingers, one period of the interdigital electrode, the aperture of the interdigital electrode, and the distance from the end of the interdigital electrode aperture to the busbar, respectively. When the aperture *W* is constant and *k*/*T* = *l*/*k* = 0.5, this type of interdigital electrode is called a uniform interdigital electrode.

Piston structure is firstly proposed by SOLAL M et al. in 2010 [17], as shown in Figure 2b, where *d*, *piston_l*, and *piston_w* represent dummy finger length, piston length, and piston width, respectively. Compared to a uniform interdigital electrode, the piston structure has added a dummy finger and a piston at the end of the aperture, based on a uniform interdigital electrode. In this structure, in order to take into account the impact of the piston on the aperture, the overlapping part of two fingers is defined as the total length of the aperture, which is the length of the aperture plus twice the piston length. The piston structure is based on the sound velocity changes in different areas of the interdigital electrode, thereby suppressing the generation of transverse modes spurious [18]. The sound velocity at the interdigital electrode gap is the highest, then the busbar area, followed by the dummy finger and aperture area, and finally the piston area. The piston structure principle is to add a slower small piston region at the edge of the aperture, resulting in a propagation mode with zero transverse wave vector in the aperture. The transverse wave vector is the real part in the slow region (represented as the gap region) and the imaginary part in the outer region (represented as the busbar region).

## 3. Resonator FEM Model and Evaluation Indicators

**Figure 3:**
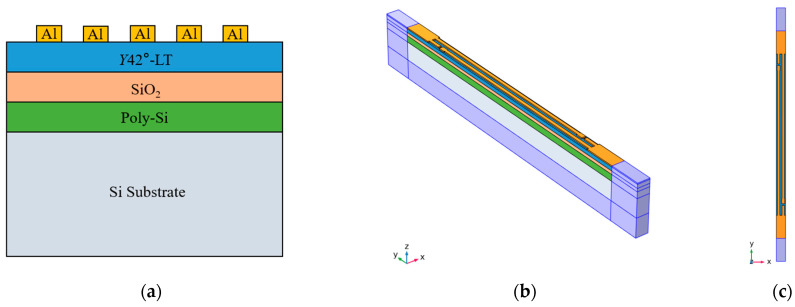
Resonator cross-sectional view and 3D FEM model. (**a**) The cross-sectional view of resonator; (**b**) overall 3D FEM model; (**c**) top view of the 3D FEM model.

The cross-sectional view of the resonator is shown in Figure 3a. The thickness of the *Y*42°-LT/SiO_2_/Poly-Si/Si substrate is 0.6 μm, 0.5 μm, 1 μm, and 350 μm, respectively; the aluminum electrode thickness is 0.18 μm. According to the above parameters, we established a 3D FEM model, as shown in Figure 3b. The specific settings are as follows: Euler angles are (0°, 48°, 0°), periodic boundary conditions are set on both sides of the propagation direction, and the perfectly matched layer (PML) is set on the bottom and both sides, respectively. The top view of the 3D FEM model is shown in Figure 3c, where the length of the busbar is 5 μm, the total aperture length is 25*T* (*T* is period), the gap length is 0.35 μm, and the length of the dummy finger is 1.08*T*.

After establishing the 3D FEM model, three commonly used performance evaluation indicators of resonators are introduced:

(1) Transverse modes spurious. When surface acoustic waves propagate in the electrode area of SAW resonators, their propagation direction is often not strictly consistent with interdigital electrodes. The vertical direction of the aperture can easily lead to transverse leakage of energy in the SAW resonator, that is, energy radiation to other areas outside the interdigital electrodes region, resulting in an increase in insertion loss and a decrease in the Q value of the device. Therefore, in the actual design of SAW resonators, the transverse electrode is often designed as a waveguide structure, causing total reflection of the energy at the boundary of the gap/interdigital electrodes region or the gap/busbar region, thereby concentrating in the central region of the interdigital electrodes. However, due to the limited aperture length of the waveguide structure, SAW resonators often excite some spurious near the center frequency, commonly known as transverse modes. The generation of transverse modes can reduce the Q value of SAW resonators, affect the performance of devices, and introduce in-band ripple to SAW filters, affecting out-of-band suppression [19,20]. 

(2) Electromechanical coupling coefficient ($K_{t}^{2}$). $K_{t}^{2}$ refers to the efficiency of energy conversion between electrical energy and mechanical energy in piezoelectric materials and is also a crucial physical quantity used to measure the strength of the piezoelectric properties of piezoelectric materials. The $K_{t}^{2}$ of the SAW resonator plays an important role in the key performance indicators of many SAW filters. The most critical one is the $K_{t}^{2}$ of the SAW resonator which determines the fractional bandwidth of the SAW filter, and the maximum relative bandwidth that the SAW filter can obtain is approximately equal to half of the SAW resonator’s $K_{t}^{2}$. For example, if designing an I.H.P. SAW filter with a relative bandwidth of 6%, it would require $K_{t}^{2}$ up to 12% of the I.H.P. SAW resonator. Evaluating the $K_{t}^{2}$ of SAW resonators can be conducted in various ways based on the measurement results [21]. The most commonly used definition is the standard definition of the Institute of Electrical and Electronics Engineers (IEEE) [22], and the IEEE standard calculation formula is:

(1) $$K_{t}^{2}=\frac{\pi }{2}\frac{fs}{fp}{\left[\tan\left(\frac{\pi }{2}\frac{fs}{fp}\right)\right]}^{-1}.$$

where *fs* is the resonant frequency and *fp* is the anti-resonant frequency. At present, the most commonly used approximate value for $K_{t}^{2}$ of SAW resonators is [23]:

(2) $$K_{t}^{2}=\frac{\pi^{2}}{4}\times (\frac{fp-fs}{fp}).$$

(3) Impedance ratio. The Q value of the SAW resonator is closely related to the insertion loss of the SAW filter; the impedance ratio is commonly used to reflect the magnitude of the Q value [24]. The larger the impedance ratio between *fs* and *fp*, the higher the Q value of the SAW resonator. The impedance formula of the resonator can be expressed as follows:

(3) $$Z=20\times \log_{10}|50\times (1+S_{11})/(1-S_{11})|.$$

where S_11_ is the voltage reflection coefficient of port 1 when resonator port 2 is matched. The impedance ratio of the resonator is the impedance of the *fp* minus the impedance of the *fs*. 

## 4. Total Aperture Length Influence

The total aperture length of the interdigital electrode is related to multiple factors such as electro-acoustic excitation efficiency, transverse modes suppression, and scattering loss. The radiation power of the interdigital electrode is proportional to its total aperture length. The longer the total aperture length, the higher the electro-acoustic excitation efficiency of the interdigital electrode, the smaller the insertion loss, and the higher the Q value of the SAW resonator. But the long total aperture length will increase the resistance loss and also produce transverse modes spurious; too small a total aperture length reduces the efficiency of interdigital electrode electroacoustic excitation and increases losses such as scattering and diffraction. Therefore, in the practical design, the total aperture length of the interdigital electrode is generally taken as 25*T*.

In the face of increasing performance demands on SAW resonators in the field of wireless communications, it is not enough to rely solely on general empirical design formulas for these structural parameters. Therefore, it is necessary to continue to optimize the structural parameters of the SAW resonator while satisfying the empirical formula.

**Figure 4:**
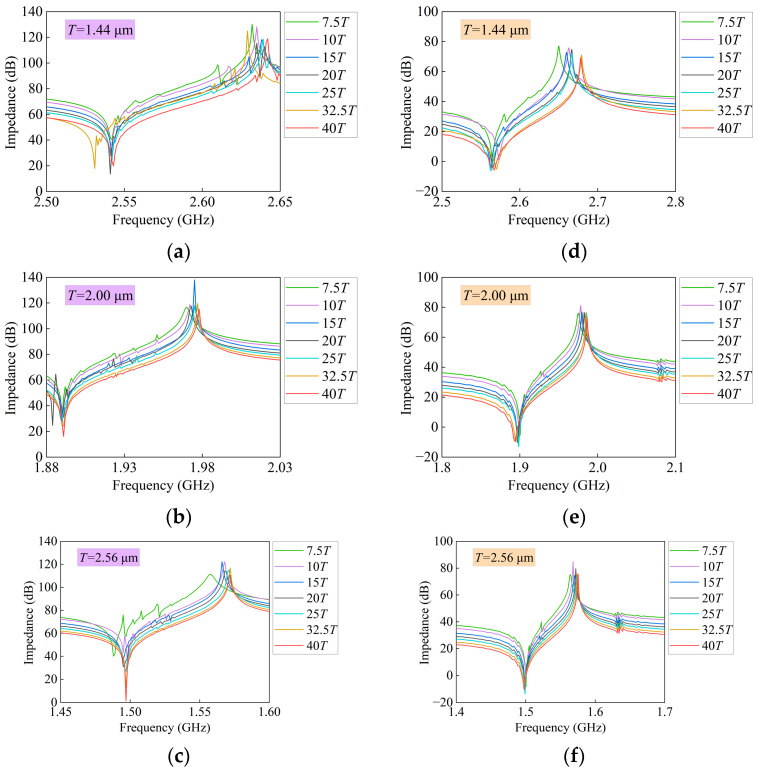
Simulation and measurement impedance curves with different total aperture lengths at different periods. (**a**–**c**) The simulation curves; (**d**–**f**) the measurement curves.

By adopting the 3D FEM model and parameters of the I.H.P. SAW resonator shown in Figure 2, we set the total aperture length as a separate variable, as shown in Table 1. We recorded different total aperture lengths at different periods as simulation parameters and performed frequency scanning to obtain a series of impedance curves regarding the impact of the total aperture length on the performance of resonators, as shown in Figure 4a–c. After photolithography, Vector Network Analyzer (VNA) was used for Ground-Signal-Ground (GSG) probe testing; the measured impedance curves of the resonators are shown in Figure 4d–f.

Firstly, the simulation results were analyzed. The transverse mode intensity between *fs* and *fp* of the SAW resonator is relatively high at the period of 1.44 μm; as the total aperture length continues to increase, the transverse modes on both the right and left sides of *fp* gradually weaken, as shown in Figure 4a. As the period increases to 2.00 μm, the transverse mode intensity between *fs* and *fp* is weaker than that at a period of 1.44 μm, but it also weakens with the increase in the total aperture length, as shown in Figure 4b. As the period increases to 2.56 μm, the transverse mode intensity between *fs* and *fp* is weaker than at periods of 1.44 μm and 2.00 μm, but it also weakens as the total aperture length increases. When the total aperture length is at least 7.5*T*, the transverse mode intensity is the strongest.

Next, we analyzed the measurement results. The *fs* position barely shifts, while the *fp* position shifts significantly, which is the same as the simulation results. What is different from the simulation results is that as the total aperture length continues to increase, the transverse modes spurious on the right side of *fs* gradually decreases. When the total aperture length is 20*T* or more, the transverse modes spurious becomes very weak until it disappears, as shown in Figure 4d. As the period increases to 2.00 μm, the *fs* of the resonator undergoes a slight shift, and the *fp* also undergoes a significant shift. As the total aperture length continues to increase, the trend of the transverse modes spurious on the right side of the *fs* is the same at 1.44 μm, as shown in Figure 4e. As the period increases to 2.56 μm, the *fs* is basically at the same position, and the offset generated by the *fp* is also relatively small. The transverse modes spurious intensity on the right side of *fs* is smaller than that at the periods of 1.44 μm and 2.00 μm, and the number of transverse modes spurious is the smallest. Similarly, as the total aperture length continues to increase, the trend of decreasing transverse modes spurious on the right side of *fs* is the same for the periods of 1.44 μm and 2.00 μm, and when the total aperture length is 20*T* or more, the transverse modes spurious also becomes weaker and disappears when it reaches 32.5*T* and 40*T*, as shown in Figure 4f. 

**Figure 5:**
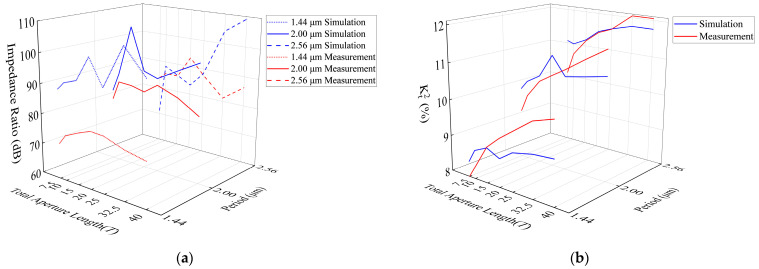
Comparison between simulation and measurement of I.H.P. SAW resonators with different periods and total aperture lengths. (**a**) Impedance ratio comparison diagram; (**b**) $K_{t}^{2}$ comparison diagram.

Finally, we analyzed the impedance ratio and $K_{t}^{2}$. Under the same period, the impedance ratio of the resonator is the highest near 20*T*; when the total aperture length is 7.5*T* or 40*T*, the impedance ratio decreases then stabilizes within a specific range and experiences slight fluctuations. In addition, the impedance ratio simulation results do not change with the change in the period, but the actual measurement results show that when the period is small, the impedance ratio decreases compared to when the period is large, as shown in Figure 5a. There are various reasons for inaccurate impedance ratio simulation results, and the most likely reason is the incorrect loss factor in the FEM model. 

Through comparison, it can be found that under different periods, when the period is 1.44 μm, $K_{t}^{2}$ is generally smaller than 2.00 μm and 2.56 μm; during the same period, when the total aperture length is 7.5*T*, $K_{t}^{2}$ is at its smallest. The $K_{t}^{2}$ gradually increases with the increase in the total aperture length, and after increasing to a certain value, there is a stable trend, as shown in Figure 5b. This is due to the limitations of the SAW resonator structure itself, which cannot be improved by infinitely increasing the total aperture length.

## 5. Piston Width and Length Influence

### 5.1. Piston Width 

**Figure 6:**
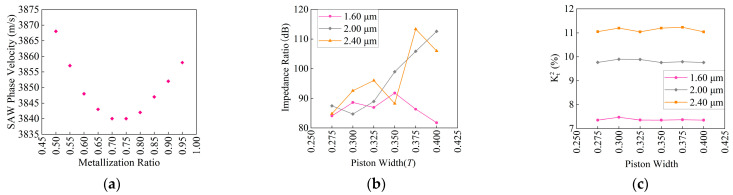
Schematic diagram of the relationship between the piston width and the SAW phase velocity, impact ratio, and $K_{t}^{2}$. (**a**) SAW phase velocity; (**b**) impedance ratio; (**c**) $K_{t}^{2}$.

In order to determine the metallization rate of the piston width, the SAW phase velocity in the piston structure under different metallization rates was calculated, as shown in Figure 6a. The SAW phase velocity first decreased and then increased as the metallization rate changed from 0.5(0.25*T*) to 0.95(0.475*T*), reaching its minimum at around 0.7(0.35*T*). The piston width and normalized velocity difference are in inverse proportion; the greater the speed difference between the piston region (slow) and the interdigital electrode region (fast), the smaller the required width of the piston region, and the smaller the width of the piston region, the smaller the impact on the overall device performance. Therefore, fixed piston length is 1*T*, and the 3D FEM model shown in Figure 3b is adopted to simulate piston width from 0.275*T* to 0.4*T*. The simulated impedance ratio, $K_{t}^{2}$ is shown in Figure 6b,c. Through the comparison in Figure 6b, it can be found that the impedance ratio ranges from 80 dB to 100 dB when the piston width is 0.275*T*–0.35*T*. As the piston width increases, the impedance ratio of the piston width at 0.375*T* and 0.4*T* continuously decreases when the resonator period is 1.60 μm; while at 2.00 μm and 2.40 μm, the impedance ratio of the piston width at 0.375*T* and 0.4*T* increases significantly. By comparing Figure 6c, it can be found that the resonator $K_{t}^{2}$ is not affected by the piston width at the same period. However, at different periods, as the period increases, $K_{t}^{2}$ becomes relatively larger. 

Considering the factors of sound velocity, impedance ratio, $K_{t}^{2}$, the interdigital electrode period, and the processing difficulty of the device comprehensively, the piston metallization rate 0.65(0.325*T*) is selected in this article for research.

### 5.2. Piston Length

**Figure 7:**
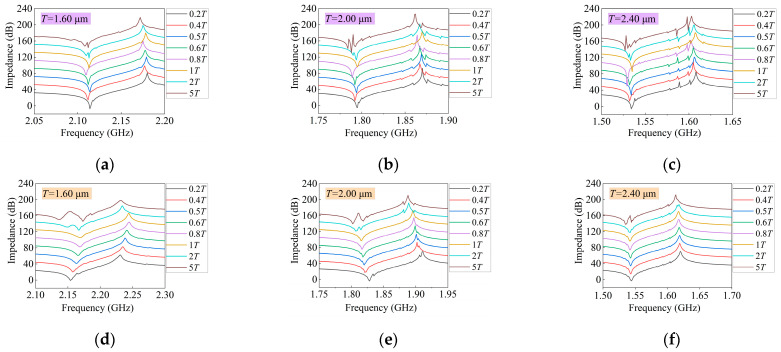
Simulation and measurement impedance curves with different piston lengths at different periods. (**a**–**c**) The simulation curves; (**d**–**f**) the measurement curves. For the convenience of observation and comparison, based on the impedance curve when the piston length is 0.2*T*, the remaining impedance curves are sequentially added by 20 dB.

For further research on the impact of piston length on the performance of the I.H.P SAW resonator, when the piston width is 0.325*T*, by applying the 3D FEM resonator model with a single pair of interdigital electrodes, as shown in Figure 3b, we manipulated the piston length as a distinct variable, as shown in Table 2. We recorded different piston lengths at different periods as simulation parameters and performed frequency scanning to obtain a series of impedance curves, shown as Figure 7a–c. After photolithography, the impedance curves of the resonators were measured and are presented in Figure 7d–f. 

By analyzing the simulation and measurement results of transverse modes spuriousness as a whole, it is evident that the intensity of the resonators’ transverse modes varies at different periods; the overall spurious intensity is more pronounced at periods of 2.00 μm and 2.40 μm than at 1.60 μm, as shown in Figure 7.

Then, we analyzed the impedance curves under different periods one by one. In Figure 7a,d, when the period is 1.60 μm, as the piston length increases, the spurious near *fs* first weakens and then strengthens, and the overall trend is the same as the simulation results. However, when the piston length is 2*T* and 5*T*, in addition to a very strong spurious on the left side of *fs*, there is no spurious near both sides of the *fp*, which is also consistent with the simulation results. In Figure 7b,e, when the period is 2.0 μm, as the piston length increases, the trend of the transverse mode near *fs* changes; it is first weakened and then strengthened when the piston length is 2*T* and 5*T*, in addition to a very strong spurious on the right side of *fs*. Near *fp*, four transverse spurious positions are changed; the measurement spurious number on the left and right side of *fp* compared to the simulation decreases from four to three. The spurious strength on the left and right side of *fp* is first weakened then strengthened, and gradually strengthened, respectively. In Figure 7c,f, when the period is 2.40 μm, transverse modes spurious intensity of the measurement results is generally weaker than those of the simulation results. As the piston length increases, the impedance curve is smooth and there is no significant spurious at a piston length from 0.2*T* to 1*T*. But a very strong spurious on the right side of *fs* is emerges and spurious intensity increases on the left side of *fp* when the piston length is 2*T* and 5*T*. 

**Figure 8:**
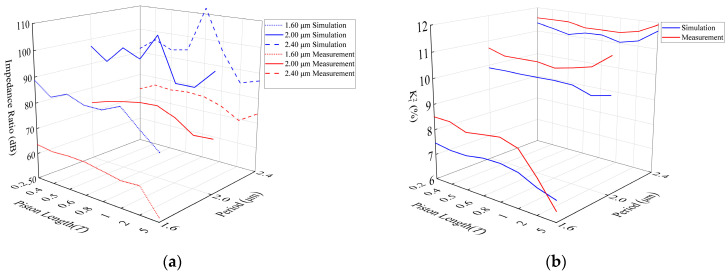
Comparison between simulation and measurement of I.H.P. SAW resonators with different periods and piston lengths. (**a**) Impedance ratio comparison curves; (**b**) $K_{t}^{2}$ comparison curves.

Finally, we analyzed the impedance ratio and $K_{t}^{2}$. In Figure 8a, through comparison, it can be found that under different periods, the impedance ratio of the resonator also increases with the increase in the periods. Both simulation and measurement results confirm this trend of change. The measured results show that the maximum impedance ratio of the resonator is 63.62 dB, 78.93 dB, and 76.67 dB when the resonator period is 1.60 μm (piston length is 0.2*T*), 2.00 μm (piston length is 0.8*T*), and 2.40 μm (piston length is 0.4*T*), respectively. Under a piston length of more than one period, when the resonator period is 1.60 μm, the impedance ratio rapidly decreases with the continuous increase in the piston length; when the resonator period is 2.00 μm and 2.40 μm, the impedance ratio slowly decreases and tends to slowly increase with the continuous increase in the piston length.

In Figure 8b, through comparison, it can be found that under different periods, the resonator $K_{t}^{2}$ also increases with the increase in the periods. At the same period, the resonator remains stable within a certain range, when the piston length is 1*T* or less, and changes significantly at 2*T* and 5*T*; the simulation and measurement results reflect these laws. When the piston length is more than 1*T* and the resonator period is 1.60 μm, it rapidly decreases with the continuous increase in the piston length. However, when the resonator period is 2.00 μm and 2.40 μm, it slowly increases with the continuous increase in the piston length.

## 6. Influence of PDT Structure on Resonator Performance

According to the previous study about piston width and length, although it is possible to reduce the transverse modes spurious and fine tune the $K_{t}^{2}$ by adjusting the piston length at different resonator periods, the impedance ratio of the resonators are all below 80 dB. A lower impedance ratio of the resonator can lead to larger insertion losses in the filter. Therefore, it is not possible to improve the impedance ratio of the resonator by changing the piston length. It is necessary to explore other microstructures to improve the performance of the resonator. 

**Figure 9:**
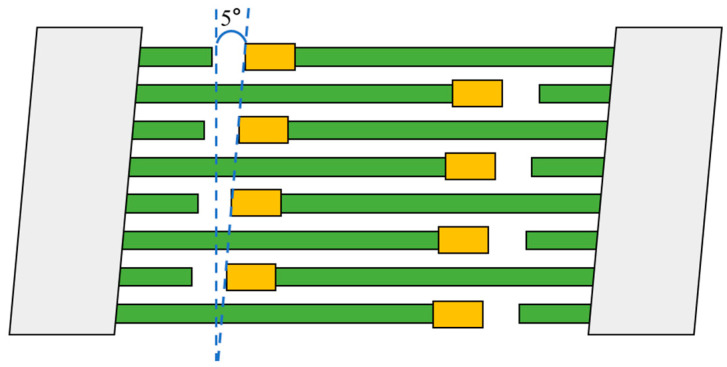
Schematic diagram of PDT structure.

In order to suppress spurious while improving impedance ratio, the PDT structure is proposed. The interdigital electrode is tilted by 5°, and the structure, after combining the dummy finger and piston structure with the tilt, is shown in Figure 9. Because the PDT structure is different from the piston and dummy finger weighted structure, it is necessary to first conduct 3D modeling of the I.H.P. SAW resonator. The single pair interdigital electrodes 3D model of the resonator can be improved on Figure 3b by tilting the interdigital electrodes. However, it is difficult to determine the periodic boundary of the 3D model with the addition of a piston. If it is necessary to simulate the overall performance of the resonator, Hierarchical Cascading Technology (HCT) needs to be used for simulation [25,26]. Considering that HCT simulation requires complex model construction and algorithm settings, in order to improve research efficiency, the resonator layout under different periods and PDT modes is directly drawn, and then, the rules are verified through actual testing.

Because the I.H.P. SAW filter often operates in the frequency range of 1 GHz to 2.5 GHz, the selected resonator periods are 1.44 μm, 1.60 μm, 2.00 μm, 2.40 μm, 2.56 μm, 2.92 μm, and 3.28 μm for verification. The interdigital electrode pairs, upper and lower reflecting grating pairs of all resonators, are 100 pairs and 15 pairs, respectively. The reflecting grating is set as a short-circuit structure. In order to adapt to the piston length and width of the resonator when studying the total aperture length, the piston length and width is set to 0.55*T* and 0.325*T*, respectively, the gap length is still fixed at 0.35 μm, and the dummy finger length is 1.08*T*.

**Figure 10:**
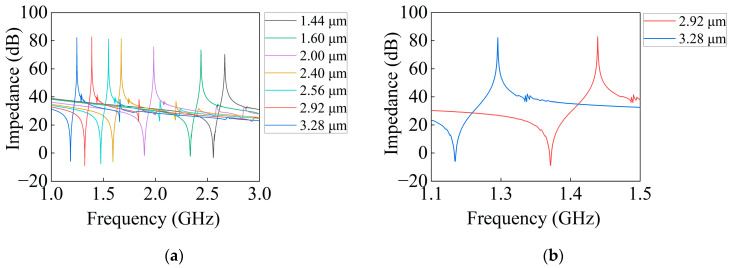
Measured impedance curves of I.H.P. SAW resonators with different periods under PDT structure. (**a**) Impedance curves at different periods; (**b**) impedance curves under periods are 2.92 μm and 3.28 μm.

After photolithography, the measured impedance curves of the resonators under the PDT structure are shown in Figure 10. It can be seen that under the PDT structure, the spurious of the resonator is well suppressed at different periods; there are no spurious near the left and right sides of *fs* and *fp*, as well as between *fs* and *fp*. Although there is a bulk wave near the right side of *fp*, it is caused by the reflecting grating and is not within the scope of interdigital electrode microstructure analysis in this study. The spurious caused by bulk waves in several locations which are also far away is not within the scope of this study.

**Figure 11:**
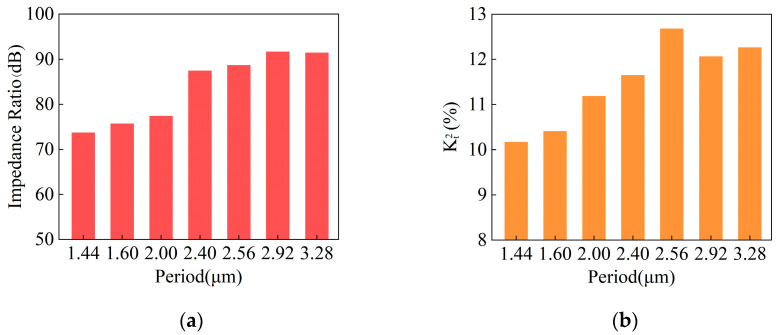
Measurement of I.H.P. SAW resonators with different periods under PDT structure. (**a**) Impedance ratio diagram; (**b**) $K_{t}^{2}$ diagram.

The measured impedance ratio and $K_{t}^{2}$ of I.H.P. SAW resonators with different periods under the PDT structure are shown in Figure 11. It can be seen that the impedance ratio and $K_{t}^{2}$ both show an increasing trend as the period increases. When the period is large, the impedance ratio and $K_{t}^{2}$ are larger. When the resonator period is 1.60 μm, 2.00 μm, and 2.40 μm, the impedance ratios are 75.74 dB, 77.43 dB, and 87.48 dB, respectively. Compared with the maximum impedance ratios of the piston structure with different piston lengths, the same period increases by 12.12 dB, −1.50 dB, and 10.81 dB, respectively. Although the impedance ratio of the resonator is basically unchanged at 2.00 μm, the impedance ratio of the PDT structure is significantly improved at 1.60 μm and 2.40 μm, and the impedance ratio of the resonator reaches over 90 dB at period is 2.92 μm and 3.28 μm. When the resonator period is 1.44 μm, $K_{t}^{2}$ is the smallest; as the period continues to increase, $K_{t}^{2}$ reaches its maximum at 2.56 μm, and as the period continues to increase, $K_{t}^{2}$ first decreases and then slowly increases.

This subsection explored the improvement of impedance ratio of I.H.P. SAW resonators at different periods through photolithography under the PDT structure. Compared with the piston structures, the impedance ratio of the resonator at periods of 1.60 μm and 2.40 μm increases by more than 10 dB, and at all periods, the PDT structure can suppress the transverse modes spurious of the I.H.P. SAW resonator. 

The PDT structure of the I.H.P. SAW resonator not only suppresses spurious and improves the impedance ratio, but also lays a solid foundation for the subsequent design of low insertion loss I.H.P. SAW filters.

## 7. GNONASS L3 Band Filter Simulation

GLONASS (GLObal NAvigation Satellite System) is a global satellite navigation system independently designed by Russia and officially launched globally in 2011. GLONASS-K1 satellite transmits CDMA signals at the new L3 frequency (1202.025 MHz); therefore, a low insertion loss and extremely narrow L3 band filter is needed to meet the GLONASS communication requirements. It is a new method which, by adopting the PDT structure in the I.H.P. SAW resonator, can meet the above needs.

It is first necessary to determine its topology circuit when optimizing extremely narrow bandwidth filters with a bandwidth of 8 MHz in Advanced Design System (ADS). Due to the shape limitation of its frequency response, the “T” type topology circuit has a relatively small insertion loss, which can achieve good suppression for the far end outside the center frequency. However, the suppression for the near center frequency is poor, making it difficult to achieve high stopband for the near frequency band. The “π” type topology circuit can achieve a wider bandwidth, with better suppression of the left and right adjacent frequency bands, but poor suppression of the far-out band.

**Figure 12:**
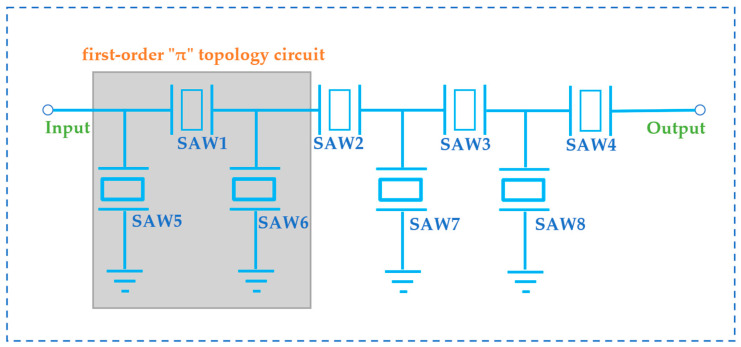
Circuit diagram of a third-order “T + π” type SAW filter.

To overcome these limitations and enhance the frequency selectivity and out-of-band suppression of L-band filters with low insertion loss and extremely narrow bandwidth (centered at 1202 MHz and an 8 MHz bandwidth), while minimizing the impact of transverse modes spurious of the resonator on in-band fluctuations, a third-order “T + π” structure is introduced. This innovative design combines the strengths of both “T” and “π” structures, as depicted in Figure 12. 

Among them, SAW1, SAW2, SAW3, and SAW4 are series resonators, SAW5, SAW6, SAW7, and SAW8 are parallel resonators. SAW1, SAW5, and SAW6 form a first-order “π” topology circuit, and the remaining series parallel resonators together form a second-order “T” topology circuit.

After determining the topology circuit structure, it is necessary to optimize the structural parameters of each resonator, such as period, interdigital transducer (IDT) number, total aperture length, etc. The main optimization objective of the band’s extremely narrow bandwidth low insertion loss GLONASS L3 filter with a center frequency of 1202 MHz and a bandwidth of 8 MHz is divided into two parts: the first part is the insertion loss within the passband, and the second part is the suppression on the passband left and right.

After several automatic optimizations by ADS and manual tuning of resonator parameters, the optimal interdigital electrode parameters for each resonator after optimization are shown in Table 3.

**Figure 13:**
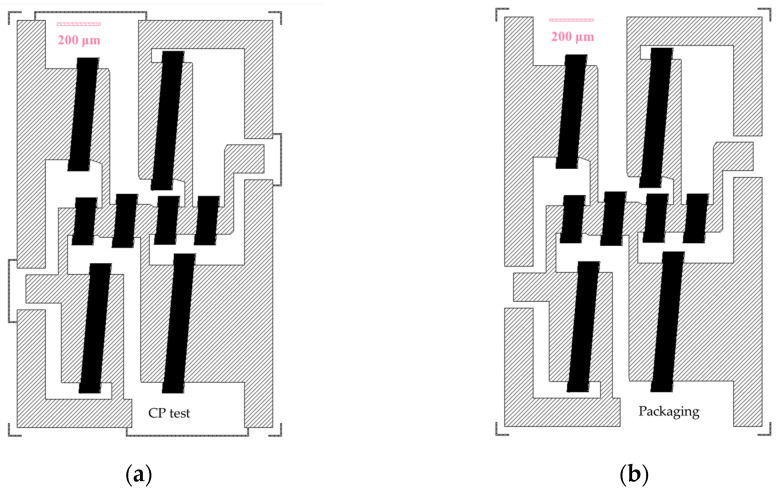
SAW filter layout. (**a**) CP test structure layout; (**b**) packaging structure layout.

Before packaging, it is often necessary to conduct chip probe (CP) testing on the filter after photolithography. If the CP test results are abnormal, the electrodes on the wafer can be cleaned and reworked to save research and development costs. Performing CP testing on the prepackaged filter can directly verify the normal operation of the filter circuit, mainly focusing on the insertion loss within the passband and can extract the S parameter of the filter for subsequent comparative analysis. However, for CP testing, it is necessary to connect the grounding terminal together to ensure signal integrity, and the impact of grounding on out-of-band suppression cannot be examined separately. By adopting open-source layout drawing software Klayout-0.27.11 for circuit layout drawing, the CP test structure layout of the SAW filter with all resonators connected is shown in Figure 13a, and the packaging layout is shown in Figure 13b. 

## 8. GNONASS L3 Band Filter Measurement

**Figure 14:**
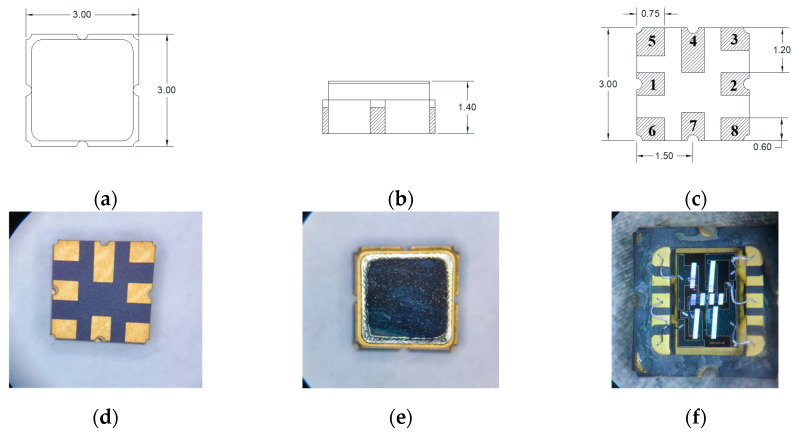
Drawing and appearance diagram of SMD 3030C packaged SAW filter. (**a**) Top view of drawing; (**b**) side view of drawing; (**c**) bottom pin diagram of drawing; (**d**) appearance of filter top view; (**e**) appearance of filter bottom pin diagram; (**f**) internal circuit diagram of SAW filter.

The GLONASS L3 band filter is packaged using a commonly used ceramic surface-mounted device (SMD) 3030 package in the industry. The SMD 3030 package is divided into Class A and Class C, and its structure is basically the same, with the only difference being the connection method of each ground terminal. Because the designed filter circuit uses four independent grounding terminals, SMD 3030C is selected for the packaging of the filter. The dimensions and pin schematic diagram of SMD 3030C are shown in Figure 14a–c. All dimensions are in millimeters, pin 2 is the RF signal input terminal, pin 1 is the RF signal output terminal, pins 3, 5, 6, and 8 are independent grounding terminals, and pins 4 and 7 are connected grounding terminals. 

After the CP test, the performance basically meets the design requirements. The device will be packaged using SMD 3030C, and the appearance after packaging is shown in Figure 14d,e. When opening the top package of SMD 3030C, the internal circuit diagram is as shown in Figure 14f. It can be observed that the internal circuit wiring is complete, and the overall appearance of the series and parallel resonators is complete.

**Figure 15:**
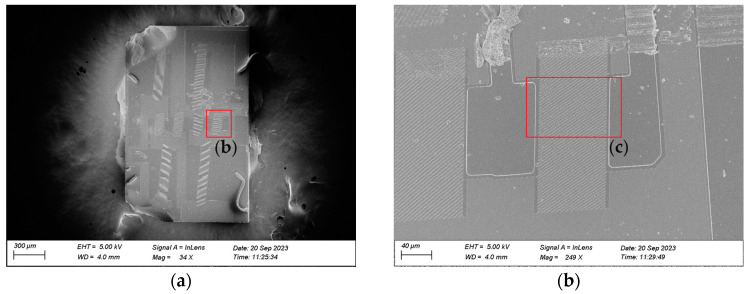

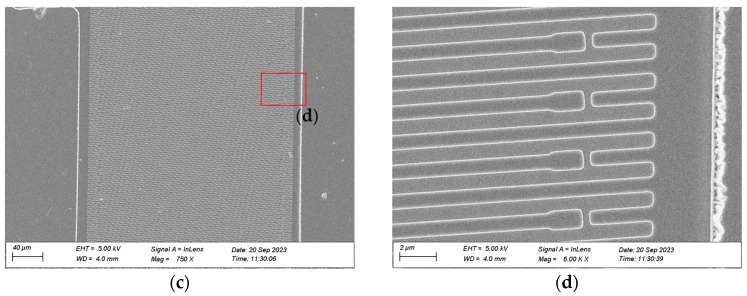
SEM image of the SAW filter and resonator. (**a**) The overall filter circuit; (**b**) the single overall resonator; (**c**) partial enlarged view of the aperture; (**d**) partial enlarged view of the PDT structure.

Figure 15 shows the scanning electron microscope (SEM) images of the I.H.P.SAW filter and resonator. As can be seen from Figure 15a–d, the overall quality of each resonator with a tilt angle of 5° is intact, which is consistent with the layout.

**Figure 16:**
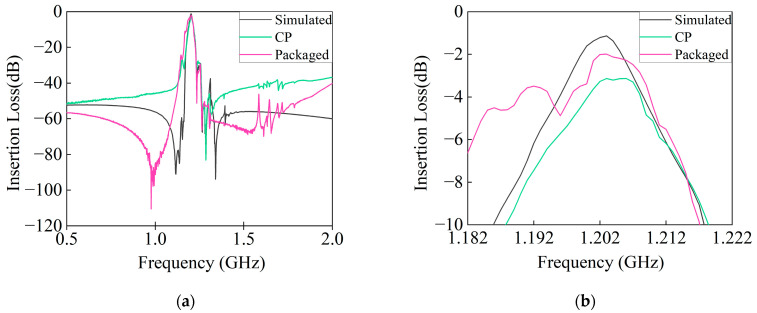
The frequency response curves of the SAW filter. (**a**) Broad band frequency response curves between 500 MHz and 2000 MHz; (**b**) narrow band frequency response curves between 1182 MHz and 1222 MHz.

After being CP tested and packaged, the measured frequency response curves of the resonators under the PDT structure are shown in Figure 16.

The center frequency of the simulated result, the CP result, and the packaged test result is 1202 MHz, 1204 MHz, and 1202 MHz, respectively. The center frequency of the CP basically reflects the simulated and packaged test center frequency; the CP results conducted a certain degree of evaluation of device performance before packaging.

The simulated and CP test results 1 dB bandwidth is 8 MHz. The packaged bandwidth increases by 1 MHz compared to the simulation and CP test results, meeting the design requirements.

Next, the main analysis focuses on the maximum insertion loss (IL_MAX_) at the center frequency and the passband. The insertion loss (IL) at the center frequency after the simulation, CP test, and package test is −1.22 dB, −3.21 dB, and −2.01 dB, respectively, while the IL_MAX_ within the 1 dB bandwidth after the simulation, CP test, and package test is −2.68 dB, −4.95 dB, and −3.73 dB, respectively. It is obvious that the IL_MAX_ is highest at the center frequency of CP within 1 dB bandwidth, which is due to the high loss at the probe, increasing the IL at the center frequency. The IL at the center frequency after packaging and the IL_MAX_ within 1 dB bandwidth are 0.79 dB and 1.05 dB higher than the simulation results, respectively, due to the introduction of additional IL in the packaging.

Finally, by comparing the results of the simulation, CP test, and packaged test, it can be found that the out-of-band attenuation of the left far end of the passband is better, the IL_MAX_ of the left far end after packaging has exceeded the design requirements, and the out-of-band attenuation of the left proximal end and the IL_MAX_ in CP and after packaging are close, but it is far lower than the designed insertion loss, which requires improving the topological circuit and increasing the electromagnetic simulation model of the package to improve the attenuation level of the left proximal end.

To enhance the suppression on the proximal right side of the passband, it becomes imperative to further reduce spurious on the right side of the resonator, particularly around the *fp* frequency. Additionally, elevating the suppression levels at the far end of the passband calls for mitigating the intensity of SAW-generated bulk acoustic waves. This can be achieved by either revising the topological circuit or modifying the resonator substrate. For a consolidated view of the simulation, CP test, and packaged test results discussed herein, refer to Table 4.

## 9. Conclusions

In this paper, a GLONASS L3 band filter with a very narrow bandwidth and low insertion loss was designed, fabricated, and tested, and the performance of resonators that play a key role in filters was studied. Through finite element simulation and actual measurement, it was determined that when the total aperture length is 20*T*, the resonator performance is better under the I.H.P. SAW substrate, and it was also found that the impedance ratio of the resonator could not be further improved by changing the piston length and width. In order to further improve the performance of the resonator, the proposed PDT structure has been experimentally proven to not only effectively suppress transverse modes spurious, but also to have a high impedance ratio.

In order to verify the performance of the resonator of the PDT structure in the filter, an extremely narrow bandwidth low insertion loss filter suitable for a GLONASS L3 band was designed and produced based on the resonator with PDT structure; the bandwidth of the filter was 8 MHz and the insertion loss was −3.73 dB in the passband. The excellent performance of the filter fully reflects the excellent performance of the resonator, not only providing a reference direction for the filter used in GLONASS satellite communication, but also providing a reference for the design of filters in similar frequency bands of BeiDou and GPS.

## Figures and Tables

**Table 1:** Simulation and test resonators: a list of total aperture length.

Period	Total Aperture Length						
	7.5*T*	10*T*	15*T*	20*T*	25*T*	32.5*T*	40*T*
*T* = 1.44 μm	*X*	*X*	*X*	*X*	*X*	*X*	*X*
*T* = 2.00 μm	*X*	*X*	*X*	*X*	*X*	*X*	*X*
*T* = 2.56 μm	*X*	*X*	*X*	*X*	*X*	*X*	*X*

*X* represents that this item has undergone simulation and actual testing.

**Table 2:** Simulation and test resonators list of piston length.

Period	Piston Length							
	0.2*T*	0.4*T*	0.5*T*	0.6*T*	0.8*T*	1*T*	2*T*	5*T*
*T* = 1.60 μm	*X*	*X*	*X*	*X*	*X*	*X*	*X*	*X*
*T* = 2.00 μm	*X*	*X*	*X*	*X*	*X*	*X*	*X*	*X*
*T* = 2.40 μm	*X*	*X*	*X*	*X*	*X*	*X*	*X*	*X*

*X* represents that this item has undergone simulation and actual testing.

**Table 3:** Optimum structural parameters of each resonator after optimization.

Resonator Number	Period	Total Aperture Length	IDT Number
SAW1	3.029 μm	41*T*	84
SAW2	3.163 μm	41*T*	96
SAW3	3.299 μm	41*T*	76
SAW4	3.458 μm	41*T*	80
SAW5	3.316 μm	41*T*	302
SAW6	3.348 μm	41*T*	252
SAW7	3.392 μm	41*T*	320
SAW8	3.216 μm	41*T*	298

**Table 4:** Results of simulation, CP test, and packaged test.

Index	Frequency Range	Simulated	CP	Packaged	Units
Center frequency	-	1202	1204	1202	MHz
1 dB bandwidth	-	8	8	9	MHz
IL at center frequency	-	−1.22	−3.21	−2.01	dB
IL_MAX_ within the passband	-	−2.68	−4.95	−3.73	dB
Passband left attenuation	500~1100 MHz	−51.29	−45.70	−57.23	dB
	1100~1150 MHz	−60.49	−26.50	−26.99	dB
Passband right attenuation	1250~1300 MHz	−30.42	−29.03	−28.85	dB
	1300~2000 MHz	−37.48	−36.74	−40.10	dB

## Data Availability

Not applicable.
